# An exploration of individual- and population-level impact of the 2-dose HPV vaccination schedule in pre-adolescent girls

**DOI:** 10.1080/21645515.2016.1160978

**Published:** 2016-05-12

**Authors:** Robine Donken, Johannes A. Bogaards, Fiona R. M. van der Klis, Chris J. L. M. Meijer, Hester E. de Melker

**Affiliations:** a Centre for Infectious Disease Control, National Institute for Public Health and the Environment (RIVM), Bilthoven, The Netherlands; b Department of Pathology, VU University Medical Center (VUmc), Amsterdam, The Netherlands

**Keywords:** antibodies, cervical Intraepithelial neoplasia (CIN), cost-effectiveness, genital warts, Human Papillomavirus (HPV) infection, human papillomavirus 16, human papillomavirus 18, immunisation schedule, non-inferiority, vaccination, transmission

## Abstract

Since 2014, several countries have implemented a 2-dose schedule for Human papillomavirus (HPV) vaccination. Licensure of the 2-dose schedule was based on non-inferiority results from immunobridging studies, comparing the antibody levels of the 2-dose schedule in young girls to those of the 3-dose schedule in young adults. Since licensure, additional data on antibody levels and other aspects of the immune response and clinical effectiveness have become available. This review will discuss the current outcomes on immunogenicity and effectiveness together with an exploration on the population impact of 2-dose schedules from a cost-effectiveness perspective. The 2-dose schedule has important benefits, such as easier logistics, reduced expenditure, potentially higher acceptance and fewer side effects. Policymakers and registration authorities should consider whether these benefits outweigh the likely differences on individual- and population-level impact between the 2- and 3-dose schedules.

## Background

Between 2006 and 2009, both the European Medicines Agency (EMA) and the Food and Drug Administration (FDA) licensed 2 prophylactic vaccines against HPV. The bivalent (2vHPV) vaccine (HPV16/18, Cervarix®, GlaxoSmithKline (GSK)) was registered for the prevention of (precursors of) cervical cancer in women. The quadrivalent (4vHPV) vaccine (HPV6/11/16/18, Gardasil®, Merck) was also indicated for the prevention of (precursors of) vaginal and vulvar cancer in females and for the prevention of (precursors of) anal cancer and genital warts in males as well as females.^1-5^ Both vaccines were initially licensed in a 3-dose schedule (0/1/6 or 0/2/6 months) for recipients starting from the age of 9 y and older. For licensure, the data on clinical efficacy in individuals above the age of 15 was used, as well as the data on immunogenicity in individuals above the age of 15 and from 9 to 14 y of age. Licensure was based on the so-called immunobridging principle,^6,7^ which assumes non-inferior clinical efficacy of a vaccine in a specific age group when the antibody levels of the vaccine in that age group are non-inferior to the antibody levels in an age group where clinical efficacy has been shown. In 2014, both vaccines were licensed in a reduced 2-dose schedule for individuals between the ages of 9 and 14 y.^8,9^ For the 4vHPV vaccine, an interval of 6 months between the first and second dose is recommended. For the 2vHPV vaccine, the second dose should be administered between 5 and 13 months after the first dose.^10^ Recently, a nonavalent (9vHPV) vaccine (Gardasil9®, Merck) became available, which protects against HPV6/11/16/18/31/33/45/52 and 58. Currently, the 9vHPV vaccine is only available in a 3-dose schedule ^11^ and is therefore beyond the scope of this review.

**Figure 1.**
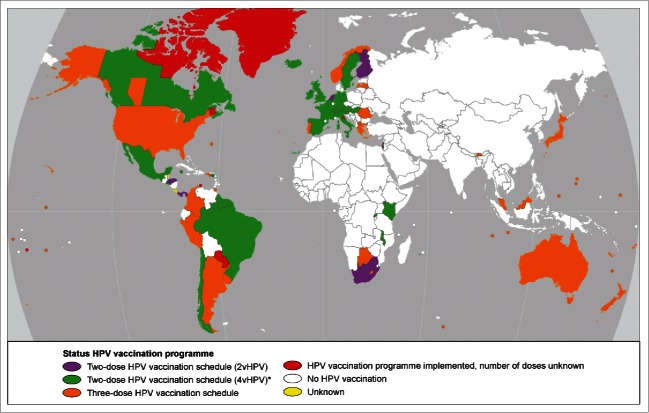
Countries that have implemented HPV vaccination in their National Immunization Program as of November 2015. Colors indicate the dosing schedules used. Primary sources used were the WHO vaccine-preventable diseases: monitoring system, HPV information center and the ECDC vaccination scheduler.^15-17^ *Five countries recommend both the 2vHPV and the 4vHPV (2-dose schedule) in their program (Kenya, Malawi, Belgium, Hungary and Italy).

Several countries have implemented the 2-dose schedule as of 2015 (Fig. 1). For the GAVI participating countries, the 2-dose schedule is recommended.^12^ The registration of the reduced dosing schedule was based on non-inferiority results from immune-bridging studies, comparing the antibody levels after a 2-dose schedule in 9- to 14-year-olds to antibody levels after a 3-dose schedule in 15- to 25-year-olds.^8,9^ In a non-inferiority study, the aim was to show that the new strategy is not worse than the currently available strategy on a particular endpoint, accepting a pre-specified marginal difference. This margin is commonly taken to be 2.0 for the antibody level ratios,^8,9,13,14^ meaning that the new dosing schedule should not induce antibody levels more than twice as low as those induced by the established dosing schedule. In the case of registration of the reduced dosing schedules for HPV vaccination, the 2-dose schedule in pre-adolescent girls did not show antibody levels that were more than twice as low as those induced by the 3-dose schedule in women older than 15 y of age, and hence non-inferiority was concluded and registration was established. Note that only between age groups were comparisons used for registration.

## Content of this review

In this review, we discuss the following important factors when considering implementing and monitoring reduced dosing schedules:

* *Immunogenicity*, both humoral and cellular immune responses,^18^ of the 2-dose schedule compared with the 3-dose schedule.
* *Effectiveness,* as measured by the occurrence of genital warts, type-specific HPV infections and cervical intraepithelial lesions (CIN), of the 2-dose schedule compared with the 3-dose schedule.
* *The potential impact*, from a population perspective, *on the transmission and cost-effectiveness* of reduced dosing schedules.

## Immunogenicity

### Antibody levels

The basis for the registration of the 2-dose schedule for individuals aged 9 to 14 y was a comparison with antibody levels after a 3-dose vaccination schedule among young adults (Table 1A and 1B).^8-10,14,19-26^ The assays most commonly used in this comparison are the competitive Luminex assay (cLIA) and the VLP-based ELISA. The ELISA assay measures the total amount of antibodies, whereas the cLIA measures a subset thereof, the neutralising antibodies for one epitope.^27,28^ Additionally, for the 4vHPV vaccine, one study used a multiplex serology assay to assess the concentration of L1-binding antibodies of HPV6, 11, 16 and 18 by measuring the median fluorescence intensity (MFI). A previous study has shown that the antibody concentrations measured using this technique are comparable to those measured using ELISA.^29^ The assay considered as the reference standard for HPV serology is the pseudovirion-based neutralisation assay (PBNA), which measures the total amount of neutralising antibodies. This assay is not often used in epidemiological studies because of its labor intensiveness.^28^ A head-to-head comparison of both vaccines administered in 2 doses among girls aged 9–14 y showed that the geometric mean antibody concentrations (GMC) as measured by ELISA were higher after the administration of the 2vHPV vaccine than after the administration of the 4vHPV vaccine. This concentration was approximately 1.7 times higher for HPV16 and 4.5 times higher for HPV18.^10^ It should be noted that higher antibody levels, up to a factor 2, were generated after a 3-dose schedule in young girls compared with the same schedule in young adults for both the 2vHPV as the 4vHPV vaccine.^30,31^ All immune-bridging studies (both 2vHPV and 4vHPV) showed higher point estimates for antibody levels after a 2-dose schedule in pre-adolescent girls (9–14 y of age) than after a 3-dose schedule in young women (15–25 y of age), except 2 studies on the 2vHPV vaccine, the HPV-070 trial (for HPV16 only) and at several time points in the studies by Romanowski (both for HPV16 and HPV18).^8,22-24^ For these studies where the 3-dose schedule in young adults generated higher antibody levels than the 2-dose schedule in young girls, the point estimates for HPV16 and HPV18 were both maximum 1.1 times higher, with confidence intervals including one. Immunobridging comparisons using a non-inferiority margin of 2.0 showed non-inferior antibody levels of the 2-dose schedule in pre-adolescent girls with the 3-dose schedule in young women, up to 60 months for the 2vHPV vaccine and up to 36 months for the 4vHPV vaccine after the first dose for HPV16 and HPV18.^10,19-24^ The only exception was the study by Krajden et al. (4vHPV), where non-inferiority for HPV16 at 36 months and HPV18 at 24 months and 36 months could not be concluded.^20^ However, in this study, the confidence intervals were large. When within-age group comparisons were made using a non-inferiority margin for the geometric mean concentration/titer (GMC/T, further on GMC) of 2.0, approximately 50% of the studies did not show non-inferior antibody levels (both 2vHPV and 4vHPV). Studies comparing the antibody levels in pre-adolescent girls (9–14 y of age) have shown 1.04–2.30 times higher antibody level for HPV18 after a 3-dose schedule, with a confidence interval that did not include 1 in more than 50% of the studies. For HPV16, there was no clear pattern; the point estimates for the antibody levels were 0.86 times lower to 2.12 times higher after a 3-dose schedule, although most point estimates after a 3-dose schedule tended to be higher. Only one study (on the 2vHPV vaccine) using PBNA for evaluation compared the total amount of neutralising antibody levels after a 2-dose schedule in pre-adolescent girls with a 3-dose schedule in young women and showed a higher or comparable total amount of neutralising antibody levels after the 2-dose schedule. In this study (2vHPV), the 2-dose schedule was also compared with a 3-dose schedule within the same age group of pre-adolescent girls. This comparison showed comparable neutralising antibody levels after 2 doses for HPV16, whereas for HPV18, the antibody levels after a 3-dose schedule were nearly twice as high than after a 2-dose schedule from 18 months post vaccination onward.^20^ Another study on the 2vHPV vaccine used PBNA to compare neutralising antibody levels within young women and showed comparable neutralising antibody levels after 2 doses for HPV16.^32^ Only one study on the 4vHPV vaccine used PBNA in addition to MFI to evaluate the neutralising titres at 18 months after the first vaccine dose among girls aged 10 to 18 y at baseline. For HPV6 and HPV16, the neutralising antibody levels were non-inferior; however, the level of neutralising antibodies for HPV18 in the 2-dose group was inferior to that in the 3-dose group, with a corresponding GMC ratio of 0.42 (0.27–0.65).^33^

**Table 1A.** Geometric mean concentrations (GMC) for HPV16-specific antibodies after 3- and 2-dose schedules and corresponding ratios at different time points. GMCs for HPV16 after 3- and 2-dose schedules as reported in several studies and corresponding ratios for dividing 3-doses by 2-doses. Based on according-to-protocol analysis population, the GMC ratio shown in this table is calculated from the GMCs, as reported in the original papers; hence, small deviations might be present from the GMC ratios, as reported in these papers.

Vaccine	Study	Age group	N	GMC 3-dose (95% CI)	N	GMC 2-dose (95% CI)	Measured at	GMC ratio (95% CI) (3-dose/2-dose)	Assay
4vHPV	Dobson^19^	9–13	251	7640 (6561–8896)	243	7457 (6388–8704)	7	1.02 (0.82–1.27)	cLIA
		16–26	246	3574 (3065–4169)			7	^*^0.48 (0.39–0.60)	
		9–13	98	1804 (1508 -2160)	96	1598 (1333–1916)	18	1.13 (0.87–1.46)	
		16–26	92	837 (695–1008)			18	^*^0.52 (0.40–0.68)	
		9–13	186	1739 (1514–1998)	195	1414 (1235–1618)	24	1.23 (1.01–1.49)	
		16–26	189	813 (709–933)			24	^*^0.57 (0.47–0.70)	
		9–13	83	1413 (1122–1780)	86	1151 (918–1444)	36	1.23 (0.89–1.70)	
		16–26	86	678 (540–850)			36	^*^0.59 (0.43–0.81)	
4vHPV	Krajden^20^	9–13	254	7332 (3641–13360)	251	8103 (3641–16318)	7	0.90 (0.34–2.44)	cLIA
		16–26	276	3641 (1808–7332)			7	^*^0.45 (0.16–1.25)	
		9–13	99	1998 (898–3641)	100	1480 (812–3641)	18	1.35 (0.48–3.77)	
		16–26	96	812 (446–1480)			18	^*^0.55 (0.21–1.43)	
		9–13	187	1808 (898–3294)	200	1480 (735–2697)	24	1.22 (0.49–3.06)	
		16–26	210	812 (446–1636)			24	^*^0.55 (0.22–1.38)	
		9–13	85	1636 (735–2981)	85	1480 (602–2441)	36	1.11 (0.41–2.97)	
		16–26	99	735 (365–1636)			36	^*^0.50 (0.18–1.39)	
4vHPV	Hernandez-Avila^26^	9–10	150	6539 (5220–8191)	145	5137 (4036–6538)	7	1.27 (0.92–1.77)	cLIA
		18–24	141	2409 (2004–2896)			7	^*^0.47 (0.35–0.64)	
		9–10	145	355 (289–435)	140	413 (338–504)	21	0.86 (0.65–1.14)	
		18–24	134	276 (226–338)			21	^*^0.67 (0.50–0.89)	
4vHPV	Sankaranarayanan^33^	10–18	1000	11 (10–12)	937	9 (8–10)	0 (day 1)	1.22 (1.06–1.41)	MFI
		10–18	308	5460 (5195–5738)	317	6125 (5785–6485)	7	0.89 (0.83–0.96)	
		10–18	313	1209 (1105–1323)	314	1222 (1116–1338)	18	0.99 (0.87–1.12)	
		10–18	271	221 (197–247)	278	163 (147–181)	36	1.36 (1.16–1.58)	
		10–18	89	218 (181–262)	127	183 (160–209)	48	1.19 (0.95–1.50)	
4vHPV	Leung^10^	9–14	322	4807 (4421–5528)	327	5056 (4597–5562)	7	0.95 (0.82–1.10)	VLP-based ELISA
2vHPV		9–14			330	8244 (7678–8852)	7		
4vHPV		9–14	315	1591 (1449–1748)	318	1285 (1151–1435)	12	1.24 (1.07–1.43)	
2vHPV		9–14			325	2218 (2023–2431)	12		
2vHPV	Lazcano-Ponce^21^	9–10	416	18219 (16833–19720)	1016	10442 (9894–11020)	7	1.74 (1.59–1.92)	VLP-based ELISA
		18–24	317	6991 (6333–7717)			7	^*^0.67 (0.60–0.75)	
		9–10	408	2376 (2216–2547)	975	1432 (1357–1510)	21	1.66 (1.52–1.81)	
		18–24	298	1035 (953–1125)			21	^*^0.72 (0.65–0.80)	
2vHPV	Romanowski 2011^24^	9–14	67	22261 (18034–27480)	65	11067 (9190–13328)	7	2.01 (1.52–2.66)	VLP-based ELISA
		15–19	60	12858 (9696–17051)	62	8442 (6895–10336)	7	1.52 (1.08–2.16)	
		20–25	51	7971 (5766–11020)	51	5673 (4377–7354)	7	1.41 (0.93–2.13)	
		15–25	111	10332 (8329–12792)			7	^*^0.93 (0.70–1.24)	
		9–14	61	3606 (2738–4750)	63	1702 (1416–2045)	24	2.12 (1.52–2.95)	
		15–25	101	1865 (1505–2311)			24	^*^1.10 (0.83–1.45)	
2vHPV	Romanowski 2014^23^	9–14			53	1595 (1298–1960)	36		VLP-based ELISA
		15–25	85	1592 (1283–1976)			36	^*^1.00 (0.74–1.35)	
		9–14			53	1320 (1084–1607)	48		
		15–25	80	1420 (1134–1777)			48	^*^1.08 (0.80–1.45)	
2vHPV	Romanowski 2015^22^	9–14			45	1369 (1104–1698)	60		
		15–25	79	1455 (1187–1782)			60		
2vHPV	Safaeian^25^	18–25	120	748 (648–867)	52	520 (422–641)	48	1.44 (1.12–1.85)	VLP-based ELISA
2vHPV	HPV-070^8^	9–14			488	9400 (8818–10020)	7		VLP-based ELISA
		15–25	352	10234 (9258–11314)				^*^1.09 (0.97–1.23)	

* Immunobridging principle. The GMC ratio for antibody levels after 3 doses (in 15- to 25-year-old women) divided by 2 doses (in 9- to 14-year-old girls).

**Table 1B.** Geometric mean concentrations (GMC) for HPV18-specific antibodies after 3- and 2-dose schedules and corresponding ratios at different time points. GMCs for HPV18 after 3- and 2-dose schedules as reported in several studies and corresponding ratios for dividing 3-doses by 2-doses. Based on according-to-protocol analysis population, the GMC ratio shown in this table is calculated from the GMCs, as reported in the original papers; hence, small deviations might be present from the GMC ratios, as reported in these papers.

Vaccine	Study	Age group	N	GMC 3 dose (95% CI)	N	GMC 2 dose (95% CI)	Measured at	GMC ratio (95% CI) (3-dose/2-dose)	Assay
4vHPV	Dobson^19^	9–13	252	1703 (1489–1946)	243	1207 (1054–1384)	7	1.41 (1.17–1.71)	cLIA
		16–26	264	661 (580–784)			7	^*^0.55 (0.45–0.67)	
		9–13	99	236 (184–304)	96	137 (106–177)	18	1.72 (1.20–2.47)	
		16–26	95	74 (57–95)			18	^*^0.54 (0.38–0.78)	
		9–13	187	267 (220–234)	195	132 (109–160)	24	2.02 (1.67–2.46)	
		16–26	202	91 (76–110)			24	^*^0.69 (0.53–0.90)	
		9–13	83	239 (175–327)	86	104 (77–141)	36	2.30 (1.49–3.55)	
		16–26	96	71 (53–95)			36	^*^0.68 (0.45–1.04)	
4vHPV	Krajden^20^	9–13	254	1808 (812–2981)	251	1212 (735–2441)	7	1.49 (0.62–3.61)	cLIA
		16–26	282	665 (299–1480)			7	^*^0.55 (0.20–1.49)	
		9–13	99	221 (110–545)	100	134 (81–270)	18	1.65 (0.61–4.49)	
		16–26	98	99 (37–148)			18	^*^0.74 (0.29–1.85)	
		9–13	187	245 (122–665)	200	148 (67–230)	24	1.66 (0.58–4.72)	
		16–26	215	110 (37–221)			24	^*^0.74 (0.25–2.20)	
		9–13	85	200 (99–545)	85	122 (55–270)	36	1.64 (0.51–5.26)	
		16–26	102	90 (33–200)			36	^*^0.74 (0.22–2.45)	
4vHPV	Hernandez-Avila^26^	9–10	150	1087 (891–1326)	145	605 (503–727)	7	1.80 (1.37–2.36)	cLIA
		18–24	141	344 (292–405)			7	^*^0.57 (0.44–0.73)	
		9–10	126	126 (105–151)	99	94 (76–115)	21	1.34 (1.02–1.77)	
		18–24	77	74 (61–89)			21	^*^0.79 (0.59–1.04)	
4vHPV	Sankaranarayanan^33^	10–18	1000	6 (5–7)	937	5 (4–5)	0 (day 1)	1.20 (0.98–1.47)	MFI
		10–18	308	2942 (2733–3167)	317	3068 (2812–3347)	7	0.96 (0.86–1.07)	
		10–18	313	377 (337–422)	314	269 (241–299)	18	1.40 (1.20–1.64)	
		10–18	271	184 (162–208)	278	117 (104–132)	36	1.57 (1.32–1.87)	
		10–18	89	206 (165–257)	127	129 (111–151)	48	1.60 (1.22–2.09)	
4vHPV	Leung^10^	9–14	333	1654 (1484–1842)	331	1207 (1093–1333)	7	1.37 (1.18–1.59)	VLP-based ELISA
2vHPV		9–14			334	5277 (4859–5732)	7		
4vHPV		9–14	326	477 (422–540)	322	264 (234–297)	12	1.81 (1.52–2.14)	
2vHPV		9–14			328	1313 (1188–1451)	12		
2vHPV	Lazcano-Ponce^21^	9–10	416	8912(8198–9687)	1016	5876 (5517–6175)	7	1.53 (1.38–1.69)	VLP-based ELISA
		18–24	317	3483 (3164–3834)			7	^*^0.60 (0.53–0.67)	
		9–10	408	1036 (952–1127)	976	619 (583–657)	21	1.67 (1.51–1.86)	
		18–24	298	438(395–485)			21	^*^0.71 (0.63–0.80)	
2vHPV	Romanowski 2011^24^	9–14	68	7399 (6033–9073)	64	5510 (4646–6535)	7	1.34 (1.03–1.75)	VLP-based ELISA
		15–19	61	4845 (3740–6277)	63	5142 (4354–6072)	7	0.94 (0.69–1.28)	
		20–25	53	3676 (2898–4664)	49	3523 (2514–4937)	7	1.04 (0.69–1.58)	
		15–25	114	4262 (3572–5084)			7	^*^0.77 (0.61–0.99)	
		9–14	63	1102 (845–1436)	63	702 (563–876)	24	1.57 (1.11–2.22)	
		15–25	103	728 (588–900)			24	^*^1.04 (0.76–1.41)	
2vHPV	Romanowski 2013^23^	9–14			52	689 (530–896)	36		VLP-based ELISA
		15–25	81	712 (560–906)			36	^*^1.03 (0.72–1.47)	
		9–14			52	543 (427–691)	48		
		15–25	79	605 (746–768)			48	^*^1.11 (0.79–1.56)	
2vHPV	Romanowski 2015^22^	9–14			43	672 (476–826)	60		
		15–25	76	635 (498–809)			60	^*^0.94 (0.65–1.36)	
2vHPV	Safaeian^25^	18–25	120	335 (285–392)	52	305 (238–391)	48	1.10 (0.82–1.47)	VLP-based ELISA
2vHPV	HPV-070^8^	9–14			493	5909 (5509–6638)	7		VLP-based ELISA
		15–25	382	5003 (4573–5473)				^*^0.85 (0.76–0.95)	

* Immunobridging principle. The GMC ratio for antibody levels after 3 doses (in 15- to 25-year-old women) divided by 2 doses (in 9- to 14-year-old girls).

### Antibody avidity

The affinity of an antibody is a measure of the strength by which one antibody binds to an antigen via a single binding site. When an antibody has a higher affinity for an antigen, the amount of antibodies needed to neutralise the antigen will be lower. The accumulated strength of multiple affinities is called avidity and may be important for protection after vaccination.^34^ Boxus et al. examined the antibody avidity of a 2-dose schedule among pre-adolescent girls (9–14 years) with the antibody avidity of a 3-dose schedule in young women (15–25 years) for the 2vHPV vaccine.^35^ No differences were found in the antibody avidity after a 2-dose schedule compared to a 3-dose schedule up to 48 months' post vaccination. Within age-group comparisons were not performed for the 2vHPV vaccine. For the 4vHPV vaccine, Sankaranarayanan et al. explored the antibody avidity after different dosing schedules among 10- to 18-year-old girls. For all vaccine types (HPV6/11/16/18), the geometric mean avidity indices after a 2-dose schedule were non-inferior to those after a 3-dose schedule, both at 7 and 18 months after the first vaccine dose.^33^

### Cellular immunity

Cellular immunity is thought to be of importance for long-term vaccine-induced protection and efficacy. T-effector cells are involved in clearance of the established infection, whereas T-memory cells are enabling B cells in providing a faster and stronger immune response. B-memory cells can differentiate into long-lived plasma cells, which are able to secrete pathogen-specific antibodies.^36,37^ An immunobridging comparison of cellular immune responses after 4vHPV vaccination was made for 2-dose schedules in girls (9–13 y of age) versus 3-dose schedules in young women (16–26 y of age). This comparison found comparable B-memory cell formation for HPV6/11/16/18, but lower memory T-cell formation for HPV6/16/18; memory T-cell formation was only comparable for HPV11. Likewise, in girls 9 to 14 y of age (4vHPV), memory T-cell formation was lower after a 2-dose schedule for HPV6/16/18, whereas a comparable response was observed for HPV11. For B-cell memory responses, no differences were found between girls (9–13 y of age) who had received 2 or 3 doses of 4vHPV vaccine.^36^ Another study comparing girls (4vHPV) within their own age group (9–14 y of age) showed similar frequencies of memory B cells and CD4+ T cells against HPV6/11/16/18 after a 2- or 3-dose schedule,^10,36^ although it should be noted that this study was not powered to compare the cell-mediated immune responses. In this study by Leung et al., a comparison was made between 2-dose recipients of the 2vHPV and 4vHPV vaccine, both between the ages of 9 and 14 y, in which the B-cells and CD4+ T-cells showed comparable frequencies for both vaccines; however, the median was highest in the 2vHPV vaccine group.^10^ These frequencies were also comparable, as reported in the study of Smolen et al.^36^ Previous research among women between 18 and 45 y of age receiving 3 vaccine doses also indicated a significantly higher proportion of memory B cells for HPV18 and for HPV16 (at 7 months and 18 months since the first vaccine dose) as well as a higher proportion of CD4+ T cells among recipients of 2vHPV compared to 4vHPV vaccine.^38^

### Summary

Non-inferior (neutralising) antibody levels and similar avidity were found for a 2-dose schedule of the 2vHPV and the 4vHPV vaccine in young girls compared with a 3-dose schedule in young adults. Comparing girls within their own age group receiving 2- or 3-doses of the 2vHPV vaccine showed non-inferior (neutralising) antibody levels in only approximately 50% of the studies. The few studies investigating memory B-cell and CD4+ T-cell formation (4vHPV) are not conclusive, and one study is not powered to investigate cellular immune response endpoints. Considering these limitations, CD4+ T-cell formation upon vaccination with the 4vHPV vaccine seems to be positively related to the number of doses given among girls (with the 3-dose schedule generating higher memory cell counts), whereas HPV18 B-cell memory seems to be affected by age (with higher age resulting in lower counts of memory B cells). It should be noted that the definition of non-inferiority is dependent on the non-inferiority margin used. Non-inferiority does not imply identical antibody levels and subsequently identical effectiveness need not be implied. The implications of the likely differences in humoral and cellular immune response on the effectiveness and duration of protection by the prophylactic HPV vaccines are unclear at the present. Consequently, vaccinated cohorts, independent of the dosing schedule, should be closely monitored. Additionally, it is presently unknown whether a new cervical HPV infection has a booster effect on the antibody levels.

## Effectiveness

The ultimate aim of HPV vaccination is to prevent cancer and ano-genital warts. Given the long time between HPV infection and the development of cancer, intermediate endpoints, such as persistent infections and (pre)cancerous lesions, are used for measuring efficacy in vaccine trials. Monitoring vaccine effectiveness to prevent persistent infections and precursor lesions in cohorts of girls who have received a 2-dose instead of a 3-dose schedule will corroborate the presently held assumption that comparable antibody levels translate into comparable protection against clinical outcomes. However, these studies are costly because they require large sample sizes and long follow-up periods. Therefore, the majority of the published data are observational and retrospective in studies not specifically designed for addressing these questions. This situation leads to methodological challenges, such as power issues and confounding, that should be considered when interpreting these data.

### Genital warts

Three-dose schedules of 4vHPV vaccine have been effective in the prevention of genital warts.^39-41^ Blomberg et al. and Herweijer et al. described the influence of alternate dosage schedules on the incidence of condyloma.^42,43^ Although the differentiation on the interval between the doses was not considered in (primary) analyses, both showed that the incidence of condyloma was significantly higher after a 2-dose than 3-dose schedule, among women up to 19 and 24 years of age, respectively. In the study by Blomberg et al., the 2-dose schedule had a higher incidence than the 3-dose schedule among women aged 24–27 years; however, this difference was not statistically significant.^42^ The incidence rate ratios (IRR) comparing the incidence after 3-dose with 2-dose schedules are shown in Table 2. Furthermore, Blomberg et al. stated that with an increasing time interval between the doses, the incidence of condyloma decreased and that this difference in IRR between 3- and 2-dose schedules was further diminished when the interval between the 2 doses increased.^42^

**Table 2.** Incidence rate ratios of 3-dose compared with 2-dose incidence rates of genital warts after 4vHPV vaccination.

Study	Interval between doses	Age group	IRR (+95% CI) (3-dose/2-dose)	Adjusted IRR (+95% CI) (3-dose/2-dose)	Adjusted for
Blomberg et al.^42^	Data for different intervals not shown. However, with increasing time between the doses, the differences between 3 and 2 doses decreased.	≤ 15 years	0.19 (0.08–0.46)	0.33 (0.13–0.85)	Age at vaccination, maternal educational level, disposable-income and calendar time
		16–17 years	0.23 (0.15–0.33)	0.35 (0.23–0.53)	
		18–19 years	0.34 (0.24–0.48)	0.48 (0.34–0.68)	
		20–21 years	0.35 (0.26–0.48)	0.49 (0.35–0.68)	
		22–23 years	0.56 (0.40–0.79)	0.59 (0.42–0.83)	
		24–27 years	0.66 (0.43–1.02)	0.56 (0.36–0.85)	
		Total	0.46 (0.39–0.54)	0.53 (0.36–0.85)	
Herweijer et al.^43^	Not considered	10–16 years	0.63 (0.43–0.93)		
		17–19 years	0.66 (0.45–0.95)		
		10–19 y (Total)	0.63 (0.48–0.82)		

### HPV infections

In the Costa Rica Vaccine trial, the effectiveness of different doses of the 2vHPV vaccine against persistent HPV16 and HPV18 infections (defined as consecutive HPV DNA detection for at least 12 months) was established in a post hoc analysis. Although the data were derived from a randomized controlled trial, the dose assignment was not randomized and the time between doses was not considered. The researchers found comparable rates of persistent infection between 3-dose and 2-dose recipients, with higher point estimates after a 3-dose schedule for HPV16/18 infection.^44^ These results were confirmed by studying the effectiveness against incident and persistent infections (defined as consecutive HPV DNA detection for at least 6 or 12 months) of vaccine types HPV16 and HPV18 in a comparison between 3-dose and 2-dose recipients in the PATRICIA trial (2vHPV), where the doses were also not randomly allocated. The point estimates for effectiveness of the 2-dose schedule were higher for a longer time interval (0/6-month schedule) between the doses compared to a 0/1-month schedule, although the findings were not significant.^45^ In an observational cross-sectional study, using cytology samples of 20- to 21-year-old women eligible for cervical cancer screening in Scotland (2vHPV), the prevalence of different HPV types by vaccination status according to different doses was assessed. The interval of the 2-dose schedule was not stated and the recommended dosing schedule at the time was the 3-dose schedule. A significant difference of 7.2% (20.8% vs. 13.6%) HPV16/18 DNA prevalence was found in women vaccinated by 2- versus 3-doses; of note, among the unvaccinated persons, the prevalence was 29.8% (95% confidence interval (CI), 28.3–31.3%).^46^ For the 4vHPV vaccine, comparable rates of incidence for vaccine types HPV6/11 and for HPV16/18 were observed after a 2-dose schedule compared to a 3-dose schedule (1.3% vs. 0.8% and 0.7% versus 0.2%, respectively).^33^ The differences in vaccine effectiveness for both 3- and 2-dose schedules against infections with HPV vaccine types are summarised in Table 3A.

**Table 3A.** Vaccine effectiveness for vaccine types HPV16 and HPV18 after 3- and 2-dose schedules compared with no vaccination after 2vHPV and 4vHPV vaccination.

Study	Vaccine	Age groups	Outcome	Proportion 3-dose (+95% CI)	Proportion 2-dose (+95% CI)	VE 3-dose (+95% CI)	VE 2-doses (+95% CI)	Proportion Two-dose (0,1 months) (+95% CI)	Proportion Two-dose (0,6 month ) (+95% CI)	VE 2-doses (0,1 month ) (+95% CI)	VE 3-doses (0,6 month) (+95% CI)
Kavanagh^46^	2vHPV	20–21 years	HPV16/18 prevalence	13.6% (11.7–15.8%)	20.8% (14.1–29.4%)	54% (46–61%)	30% (−1–52%)				
Kreimer – Costa Rica Vaccine Trial^44^	2vHPV	18–25 years	12 month (incident) persistent HPV16/18 infection	0.85% (0.56–1.2%)	0.71% (0.18–1.9%)	81% ^1 ^(71–87%)	84% ^1 ^(46–95%)				
Kreimer – Costa Rica Vaccine Trial/PATRICIA trial (modified total vaccinated cohort)^45^	2vHPV	15–25 years	Incident detection HPV16/18	Rate per 100 PY 1.23 (1.12–1.34)	Rate per 100 PY 0.87 (0.56–1.29)	77% ^1^ (75–79%)	76% ^1^ (62–85%)	Rate per 100 PY 0.90 (0.49–1.53)	Rate per 100 PY 0.68 (0.17–1.85)	75% (54–88%)	83% (42–96%)
			Incident HPV16/18 detection, persisting for at least 6 months	Rate per 100 PY 0.26 (0.22–0.31)	Rate per 100 PY 0.16 (0.05–0.38)	89% ^1 ^(87–91%)	90% ^1 ^(73–97%)				
			Incident HPV16/18 detection, persisting for at least 12 months	Rate per 100 PY 0.19 (0.15–0.24)	Rate per 100 PY 0.12 (0.03–0.32)	87% ^1^ (84–90%)	90% ^1 ^(69–98%)				
Sankaranarayanan^33^	4vHPV	10–18 years	Incident detection HPV16/18	0.4% (0.0–1.3%)				1.3% (0.6–2.4%)	0.8% (0.2–1.9%)		
			Incident detection HPV6/11	0.2% (0.0–1.0%)				0.7% (0.2–1.6%)	0.2% (0.0–1.0%)		

PY, person years. ^1^Both vaccinated groups were compared to their own control group, composed of participants randomized for the control vaccine (Hepatitis A) who had received an equal number of doses.

The Scottish study by Kavanagh et al. also explored the effect on cross-protective HPV types 31/33/45.^46^ No significant differences were found for the prevalent detection of cross-protective HPV types 31/33/45 combined, although the prevalence estimate (for all types combined) was slightly higher after a 2-dose schedule (6.8% and 7.5%, respectively, compared among the unvaccinated 13.1%).^46^ The effect of dosing schedule on cross-protective HPV types was also assessed in the PATRICIA trial (2vHPV). The effectiveness against incident infection by HPV31/33/45 (combined) was significantly higher after a 3-dose than after a 2-dose schedule: 59.7% (95% CI, 56.0–63.0%) and 37.7% (95% CI, 12.4–55.9%), respectively. Additionally, the 3-dose schedule showed significant effectiveness against 6- or 12-month persistent infection by HPV31/33/45 (combined), respectively, 60.1% (95% CI, 54.0–65.4%) and 54.9% (95% CI, 46.2–62.3%) but not for the 2-dose schedule, respectively, 30.7% (95% CI, -27.9–63.0%) and 7.6% (-118–61%). The stratification for the timing of the second dose yielded a vaccine effectiveness of the 2-dose schedule (0/6 months) that was significantly higher than after a 2-dose schedule (0/1 month) against infections that persisted for at least 6 months with HPV31/33/45 combined.^45^ For the 4vHPV vaccine, no differences in incidence for the cross-protective types HPV31/33/45 could be observed between the 3- and 2-dose (both 0/1 and 0/6 month intervals) schedules.^33^ The vaccine effectiveness for both 3- and 2-dose schedules against infections with cross-protective types are summarised in Table 3B.

**Table 3B.** Vaccine effectiveness for cross-protective types HPV31, HPV33 and HPV45 after 3- and 2-dose schedules compared with no vaccination after 2vHPV and 4vHPV vaccination.

Study	Vaccine	Outcome	Proportion 3-dose (+95% CI)	Proportion 2-dose (+95% CI)	VE 3-dose (+95% CI)	VE 2-dose (+95% CI)	Proportion Two-dose (0,1 month) (+95% CI)	Proportion Two-dose (0,6 month) (+95% CI)	VE 3-dose (0,1 month) (+95% CI)	VE 3-dose (0,6 month) (+95% CI)
Kavanagh^46^	2vHPV	HPV31/33/45 prevalence	6.8% (5.5–8.5%)	7.5% (3.9–14.2%)	85% ^1^ (81–88%)	83% ^1^ (66–91%)				
Kreimer – Costa Rica Vaccine Trial/ PATRICIA trial (modified total vaccinated cohort)^45^		2vHPV	Incident one-time detection of HPV31/33/45	Rate per 100 PY 1.65 (1.53–1.78)	Rate per 100 PY 2.21 (1.68–2.85)	59.7% ^1^ (56.0–63.0%)	37.7% ^1^ (12.4–55.9%)				
			Incident detection of HPV31/33/45 that persisted at least 6 months	Rate per 100 PY 0.61 (0.54–0.69)	Rate per 100 PY 0.71 (0.43–1.09)	60.1% ^1^ (54.0–65.4%)	30.7% ^1^ (-27.9–63.0%)	Rate per 100 PY 2.76 (1.96–3.78)	Rate per 100 PY 3.07 (2.21–4.18)	10% (−42–43%)	68% (27–87%)
			Incident detection of HPV31/33/45 that persisted at least 12 months	Rate per 100 PY 0.40 (0.34–0.46)	Rate per 100 PY 0.43 (0.23–0.74)	54.9% ^1^ (46.2–62.3%)	7.6% ^1^ (−118 - 61%)				
Sankaranarayanan^33^	4vHPV	Incident detection HPV31/33/45	6.0% (4.1–8.3%)				4.6% (3.2–6.4%)	4.9% (3.3–7.2%)			

PY, person years. ^1^Both vaccinated groups were compared to their own control group, composed of participants randomized for the control vaccine (Hepatitis A) who had received an equal number of doses.

### Cervical lesions

To date, evidence on the effectiveness of the 2-dose schedule (compared with the 3-dose schedule) against cervical precursor lesions is based on 4 data-linkage studies (3 4vHPV vaccine and one 2vHPV vaccine), which have determined the association between vaccine status and the prevalence of cervical intraepithelial neoplasia (CIN). Three of these 4 studies were performed in Australia (4vHPV vaccine), the first country to adopt a nationwide female immunisation program. All four studies found differences in the risk estimates for high-grade CIN, with a higher, although not significant, risk after a 2-dose schedule.^47-50^ Only the study by Brotherton et al. showed statistically significant differences with a higher risk for a 2-dose schedule (Table 4).^47^ However, these studies were not designed for a specific 3- vs. 2-dose comparison. Methodological challenges in all 4 studies were that confounding could not be completely accounted for and that these studies were not powered to detect a statistically significant difference. Notably, all of the studies reported comparable point estimates; however, these findings were only significant in the study with the largest sample size. It should also be noted that parts of the screened populations participated in a catch-up program; thus, these women were vaccinated above the age of fourteen. Presently, for the recipients older than 14 y of age, the 3-dose schedule is still recommended. The studies by Brotherton et al. and Crowe et al. mentioned a likely misclassification with regard to underreporting the number of received doses in the vaccination registries, which might lead to overestimation of the effect of partial vaccination.^47,48^

**Table 4.** Vaccine effectiveness (VE) of different dosing schedules on cervical precursor lesions.

Study	Vaccine	Design	Age groups	Endpoint	Effect estimate	Adjusted for	Adjusted effect estimate 3-doses^*^	Adjusted effect estimate 2-doses^*^
Brotherton et al. (Vaccination before first screen) ^46^	4vHPV	Data-linkage	12–26 years	Any high grade	Hazard Ratio	Age in 2007, remoteness and SES	0.71 (0.64–0.80)	1.21 (1.02–1.44)
				CIN3/AIS			0.69 (0.58–0.81)	1.17 (0.92–1.48)
				CIN2			0.75 (0.65–0.86)	1.22 (0.97–1.54)
				High-grade cytology			0.53 (0.47–0.60)	0.63 (0.50–0.80)
				Low-grade cytology			0.73 (0.68–0.78)	0.52 (0.44–0.61)
Crowe et al. (all ages) ^47^	4vHPV	Data-linkage	11–27 years	High-grade cases	Odds Ratio	SES, remoteness, year of birth, quartile of follow-up times	0.54 (0.43–0.67)	0.79 (0.64–0.98)
				Other cases			0.66 (0.62–0.70)	0.79 (0.74–0.85)
Gertig et al. ^48^	4vHPV	Data-linkage	12–17 years	Any high grade	Hazard ratio	Remoteness, SES and age at first screening	0.61 (0.48–0.78)	1.02 (0.68–1.53)
				CIN3/AIS			0.53 (0.36–0.77)	0.87 (0.46–1.67)
				CIN2			0.70 (0.52–0.94)	0.99 (0.59–1.64)
				CIN1			0.82 (0.66–1.0)	0.90 (0.61–1.33)
				High-grade cytology			0.71 (0.61–0.83)	0.95 (0.73–1.23)
				Low-grade cytology			0.79 (0.75–0.84)	0.64 (0.57–0.72)
Pollock et al. ^49^	2vHPV	Data-linkage	20–21 years	CIN 3	Risk Ratio	Cohort year, deprivation score and age in months	0.45 (0.35–0.58)	0.77 (0.49–1.21)
				CIN 2			0.5 (0.40–0.63)	0.81 (0.54–1.22)
				CIN1			0.71 (0.58–0.87)	0.65 (0.42–1.01)

* The reported effect estimates are relative to the unvaccinated. A ratio below 1 indicates a protective effect of vaccination on cervical precursor lesions. The closer the effect estimate is to 0, the lower is the risk for a cervical precursor lesion.

### Summary

The data from within age group comparisons on genital warts (4vHPV vaccine only), incident and persistent HPV infections (both 2vHPV and 4vHPV vaccine) and cervical lesions (both vaccines) are derived from post hoc analyses of non-randomized or observational studies. These studies indicated in several cases a lower effectiveness for a 2-dose schedule. The incidence of genital warts after a 3-dose schedule was found to be lower than after a 2-dose schedule for 4vHPV vaccine, although the timing between the doses might have been suboptimal.^42,43^ The vaccine effectiveness for incident and persistent HPV16/18 infection was comparable between 3-and 2-dose schedules of 2vHPV vaccine in non-randomized subgroup analyses from vaccine trials.^44-46^ After a 2-dose schedule, no significant cross-protection against persistent infections with types HPV31/33/45 was found, although when the timing of the 2-dose schedule was (0,6 months), the results were comparable to the 3-dose schedule.^45^ For the 4vHPV vaccine, no differences between the 3- and 2-dose recipients were observed in the incidence of persistent infections with vaccine types HPV6/11/16/18 or cross-protective types HPV31/33/45.^33^ Several studies on cytological abnormalities showed higher but not significant risk estimates for high-grade CIN after a 2-dose schedule. In only one study, a significant difference was found.^47-50^

## Population perspective

### Impact on transmission

Until now, we have only considered the effects of reduced dosing schedules on the level of the vaccinated individual. The overall impact of a vaccination program, however, consists of direct protection against infection together with indirect protection through a reduced circulation of vaccine-preventable pathogen strains. The indirect protection of non-vaccinated individuals by vaccination is called herd immunity. Herd immunity may constitute an important aspect of the overall impact of a vaccination program, as demonstrated by the rapid decrease in the diagnosis of genital warts in countries with a substantial uptake of the 4vHPV vaccine.^39-41^ The prevalence of genital warts declined among the vaccinated (females) as well as non-vaccinated (heterosexual) individuals, likely as a consequence of reduced exposure to vaccine-type infections in the heterosexual population as a whole. Modeling studies have also predicted substantial herd immunity for oncogenic HPV types ^51,52^, although the magnitude of indirect protection will likely be smaller for HPV16 and 18 relative to HPV6 and 11.^53^ Although herd immunity from HPV vaccination is becoming measurable, for example, by the reduced prevalence of genital warts, the mechanisms that underlie a reduced circulation of vaccine-type HPV are not precisely clear. The prophylactic efficacy of HPV vaccines approximates 100% when the clinical endpoints, i.e., vaccine-type-specific high-grade lesions or genital warts, are considered. However, the efficacy is lower when prevalent detection of HPV DNA is considered.^54,55^ Whether single HPV-DNA detection using a highly sensitive PCR should be classified as infection or merely as exposure is open to discussion; however, it is conceivable that vaccinated individuals might become transiently infected by vaccine-type HPV. This situation, for example, is illustrated in the study by Kavanagh et al., where HPV16/18 was detected among the vaccinated individuals, although still at a lower rate than among the unvaccinated individuals.^46^ Moreover, HPV16/18 prevalence was higher among those vaccinated with a 2-dose compared with a 3-dose schedule.^46^ To what extent such transient infections still contribute to further transmission remains unclear, but it is likely dependent on viral load. If non-inferiority in antibody levels induced by 2-dose versus 3-dose vaccination schedules would result in similar protection against clinical endpoints on an individual level, this situation need not translate into similar transmission dynamics, e.g., if differences exist in the duration of transient infections among those vaccinated with 3- or 2-doses. The population-level impact of reduced dosing schedules on HPV transmission dynamics have yet to be investigated. Modeling studies have so far only been employed to assess the impact of differences in the duration of protection induced by 2- vs. 3-dose vaccination schedules. The study of Jit et al. showed that when the 2-dose schedule protects for at least 20 y, the effects of the third dose are small because those vaccinated will have aged beyond the peak of sexual transmission rates.^56^ Additionally, when the coverage is 80% and protection of the 2-dose schedule lasts for 20 y, the numbers needed to vaccinate with the 3-dose schedule to prevent one additional case of cervical cancer exceed 10,000.^57^

### Cost-effectiveness of reduced dosing schedules

The 2-dose schedule could have important benefits, such as lower costs, higher uptake, higher completion rates, easier logistics and likely fewer adverse events.^24^ The reduced expenditure is an obvious advantage when switching to reduced dosing schedules for HPV vaccination. In health economic terms, it can be considered to be a justification of a potentially reduced population impact of vaccination. From the dynamic modeling study of Jit et al., one might already expect that if a 2-dose vaccination would provide the same protection as 3-dose vaccination for 20 y, then the third dose will need to be priced substantially lower for a 3-dose schedule to be cost-effective, as illustrated in a separate cost-effectiveness analysis for the UK assuming a single-dose price of approximately £85.^57^ Laprise and colleagues have assessed the incremental cost-effectiveness of a third vaccine dose on top of a 2-dose schedule for Canada, assuming a dose price of $85, and they concluded that a third vaccine dose is unlikely to be cost-effective if 2 doses achieve similar protection for 30 y or longer.^58^ It should be mentioned that the realized vaccine prices in a national immunisation program are typically lower than the pharmacy dose prices used in cost-effectiveness analyses, which could have affected the outcomes of these studies.

### Summary

The cost-effectiveness analyses of the 2- versus 3-dose HPV vaccination schedules for the UK and Canada achieved similar results, partly because they started from similar assumptions. Both studies were based on the principle that the 2-dose vaccination achieves identical protection against infection as a 3-dose vaccination schedule but might achieve a shorter duration of protection. The assumption that non-inferiority of antibody levels from immune-bridging studies translates into identical effectiveness on an individual level, and identical impact on secondary transmission might not hold because there are indications that the ratio of antibody levels between 3- and 2-dose schedules is not constant over time.^14^ Moreover, based on observational studies, one might conclude a reduced effectiveness from a reduced dosing schedule.^42,43,46,47^ The effectiveness that needs to be achieved by 2- vs. 3-dose vaccination for health economic preference of either vaccination schedule warrants additional exploration.

## Concluding remarks

Since licensure in 2013/2014, several countries worldwide have implemented a 2-dose schedule for HPV vaccination. The two-dose schedule has important benefits compared to the 3-dose schedule, such as easier logistics, potentially higher uptake and fewer side effects. Although immunogenicity for 2- versus 3-dose schedules seems unimpaired when pre-adolescent girls are compared to young adults, non-inferiority within the same age group has not been shown. Additionally, the non-inferiority studies performed so far have a short follow-up, the longest time frame being only 48 (4vHPV) or 60 months (2vHPV). Comparable immunogenicity of the 2-dose compared with 3-dose schedule should be safeguarded over a longer time frame in prospective studies, preferably until the girls who are vaccinated with a 2-dose schedule become eligible for cervical screening. Some differences in the effectiveness on the prevalence of HPV16/18 infections and persistence of cross-protective types HPV31/33/45 have been found, and indications for an increased risk for CIN2/3 after a 2-dose schedule exist. So far, most results on the effectiveness of the 2-dose relative to a 3-dose schedule were obtained using post hoc analyses in studies not designed or randomized for evaluation of these outcomes, did not differentiate on timing between doses, could not completely account for confounding or were not powered to detect statistically significant differences. Hence, additionally studying the effectiveness in long-term prospective studies against persistent infections and CIN2+ of reduced dosing schedules, eventually compared to a 3-dose schedule, is of additional value. The impact of reduced dosing schedules on transmission dynamics and herd immunity will become clear in the following years, with more countries implementing 2-dose schedules. Thus far, the cost-effectiveness analyses have only considered a shorter duration of protection, but with an equal effectiveness of the 2-dose schedule relative to the 3-dose schedule. Moreover, health economic assessments were based on vaccine prices that were higher than might be applicable in national immunisation programs. These analyses showed that with at least 20 years' protection of the 2-dose schedule, the incremental benefits of the 3-dose schedule are small. Other scenarios for cost-effectiveness evaluation must be explored in the future. Long-term follow-up of cohorts vaccinated with a 2-dose schedule on both immunogenicity and effectiveness is indicated. These studies might also help to elucidate a likely correlate of protection.
